# Health Workforce Attrition in Lithuania: Migration and Career Exit Intentions

**DOI:** 10.3390/healthcare13192470

**Published:** 2025-09-29

**Authors:** Linas Šablinskas, Mindaugas Stankūnas

**Affiliations:** Department of Health Management, Lithuanian University of Health Sciences, 44307 Kaunas, Lithuania; mindaugas.stankunas@lsmu.lt

**Keywords:** health workforce, emigration of physicians, career intentions, health policy, reasons for migration, Lithuania

## Abstract

**Background/Objectives**: Human resources for health remain one of the key factors ensuring the effectiveness and accessibility of the healthcare system. Many countries face a shortage of physicians due to the migration of healthcare professionals and career changes, making it crucial to understand the underlying causes of these processes. The aim of this study was to assess the intentions to emigrate among Lithuanian medical students, residents, and practicing physicians, as well as to identify the main reasons for migration and plans regarding employment in the medical profession. **Methods**: The study was conducted using an anonymous, author-designed questionnaire survey, in which 1367 respondents participated. **Results**: The results indicate that 50.91% of students, 39.70% of residents, and 36.81% of physicians plan to emigrate or do not intend to work in the medical profession at all. The main reasons specified for emigration were higher salaries, better living conditions, and greater professional opportunities. The primary reasons for leaving the medical profession included low salary, physical and psychological strain, and administrative burden (among physicians), also the perception that the profession is not suitable for them (among students and residents). **Conclusions**: These findings are important for shaping human resource policies and strategies in the Lithuanian healthcare system and may also be valuable for policymakers in other countries facing similar challenges.

## 1. Introduction

11 March 1990, following the restoration of independence, Lithuania initiated significant public sector reforms, including in the healthcare system. As in many other post-communist countries, Lithuania had inherited the Semashko model of healthcare organization, which was characterized by a centralized planning system, universal access to healthcare services, a hospital-centered structure, and the absence of a private healthcare sector [1,2].

As the Lithuanian healthcare system underwent fundamental reforms aimed at improving population health, the quality of services provided, and ensuring patient choice, greater attention was directed toward the development of primary healthcare and the training of general practitioners. Furthermore, the National Concept of Health for Lithuania, approved by the Lithuanian parliament, established the priorities for healthcare system reform and became the foundation for the formation of Lithuania’s health policy [3,4].

The quality and accessibility of healthcare services are closely linked to human resources—particularly the number, qualifications, distribution, and motivation of physicians. A shortage of healthcare professionals is a growing global concern affecting both developed and developing countries, and this issue has become even more pronounced in recent years due to the COVID-19 pandemic [5,6]. The consequences of the pandemic not only increased the workload of healthcare workers but also exposed long-standing structural weaknesses in healthcare systems, particularly in the areas of workforce management and well-being [7].

Physician migration is one of the key contributors to the shortage of medical professionals across nations. International migration has become a global trend, driven by a combination of structural, economic, and personal factors. According to the World Health Organization (WHO), the movement of healthcare workers from less-developed to more-developed countries poses a significant threat to the global sustainability of healthcare systems [8]. This migration often results in a “double loss” effect, whereby countries not only lose their investment in medical education but also face reduced capacity to provide quality healthcare services [9].

The factors influencing migration can be broadly categorized into “push” and “pull” factors [10]. Push factors typically include poor working conditions, inadequate salaries, excessive workload, limited career prospects, lack of professional development opportunities, emotional burnout, and poor work–life balance [11]. Pull factors, on the other hand, are usually related to better economic conditions, social security, professional recognition, and the opportunity to work in modern, technologically advanced healthcare systems [12].

In the European context, the migration of healthcare professionals particularly affects Central and Eastern European countries, including Lithuania. Research indicates that physician migration is a persistent issue, with a significant proportion of young professionals expressing their intention to work abroad either during or shortly after completing their studies [13]. This trend contributes not only to the loss of trained professionals but also to reduced access to healthcare services in smaller towns and rural areas, where shortages are already evident [14].

When developing human resources strategies for the healthcare system, it is important to consider not only emigration but also the attrition of physicians from the profession altogether. This issue became especially evident during the COVID-19 pandemic, when high workloads and continuous stress led some healthcare workers to leave the system [15,16,17]. Although the pandemic triggered the most significant wave of departures, the process remains ongoing and continues to affect the number of practicing physicians.

Broadly defined, health workforce attrition refers to the reduction in the number of professionals within the system due to their withdrawal from the medical profession or the labor market altogether. Attrition can be categorized into two main types: voluntary attrition, where individuals independently decide to leave (e.g., due to burnout, emigration, or career change), and involuntary attrition, where the exit is driven by external circumstances (e.g., health problems or systemic changes) [18,19]. It is also important to distinguish between internal attrition, in which the individual remains within the healthcare sector but changes position or institution, and external attrition, which involves a complete departure from the medical field, either domestically or through emigration [20].

To assess the risk of attrition, research often relies on the indicator of career exit intention, defined as a person’s subjective decision or inclination to leave the medical profession in the near or more distant future. Studies have shown that such intentions may serve as significant early predictors of actual workforce withdrawal, particularly among younger professionals [19,21]. These intentions are typically shaped by a combination of psychological factors—such as burnout, emotional exhaustion, and uncertainty about professional identity—and labor market conditions, including inadequate remuneration, excessive workloads, limited opportunities for advancement, and unsatisfactory working environments.

An additional challenge is posed by physician migration, which—although it does not necessarily imply professional exit—often has similar consequences at the national level. Physicians who emigrate in search of better opportunities abroad typically continue practicing medicine but no longer contribute to the healthcare system of their home country. In smaller nations such as Lithuania, even moderate losses of qualified professionals may have a disproportionately large impact on the system’s capacity and accessibility.

Although attrition, migration, and career exit intentions are often viewed as related phenomena, they are not identical. Attrition is the outcome; migration is a geographical and institutional transition, often driven by economic or systemic push-pull factors; and career exit intention is a psychological decision, which may—but does not necessarily—translate into actual behavior [22,23]. Distinguishing among these processes is essential for accurately assessing health workforce risks, identifying the most vulnerable groups, and developing data-informed human resource policies.

Despite growing awareness of the problem, there is still lack of data at the national level regarding the motivations, expectations, and intentions behind physician migration. Therefore, it is essential to understand how migration is perceived by medical students, residents, and practicing physicians—both future and current participants in the healthcare system. The aim of this study is to analyze the intentions to migrate or leave the profession among Lithuanian medical students, residents, and physicians, as well as the motivational factors driving these decisions. The findings may contribute to the development of effective health workforce policies and long-term retention strategies in Lithuania and similar countries.

## 2. Materials and Methods

Data for this study were collected in Lithuania between 2022 and 2023, involving a questionnaire survey with medical students, medical residents, and physicians, with a total of 1367 participants. The data collection methods and sampling procedures varied slightly among the respondent groups.

*Medical students.* Lithuania has two medical training schools: the Lithuanian University of Health Sciences (LSMU) and Vilnius University (VU). Data was collected using an online questionnaire developed in Microsoft Forms. The survey link and QR code were distributed via university-provided institutional email addresses. To further encourage participation, at the Lithuanian University of Health Sciences (LSMU), and with prior agreement from lecturers, students were shown the survey’s QR code during lectures and invited to complete the questionnaire. The survey was sent to 5th- and 6th-year medical students at both LSMU and VU who were studying in Lithuanian at the time of data collection, including Lithuanian students enrolled in mixed groups alongside international students. The survey link and QR code were not sent to students who were on academic leave during the data collection period or to international students. A total of 497 medical students participated in this survey (this sample represents 58.75% of Lithuanian 5th and 6th year medical students).

*Medical residents*. Data were collected using an online questionnaire developed in Microsoft Forms, which was distributed to medical residents at LSMU and VU who were actively enrolled in residency programs at the time of the survey. The survey link and QR code were shared via institutional email addresses provided by the universities. Additionally, residency program representatives were invited to disseminate the survey link and QR code among their peers. Residents on academic leave during the data collection period did not receive the survey. A total of 199 medical residents participated in this survey (this sample represents 12.33% of Lithuanian medical residents).

*Physicians*. Data was collected using an online questionnaire developed in Microsoft Forms. The survey link and QR code were distributed to Lithuanian healthcare institutions that have contracts with the National Health Insurance Fund under the Ministry of Health. Heads of these institutions were asked to share the survey link and QR code with physicians working within their facilities. To increase physician engagement, the Ministry of Health of the Republic of Lithuania also sent out a request to healthcare institutions encouraging them to disseminate the questionnaire. The survey was completed by physicians who were employed at the time of the study in these healthcare institutions and were fluent in Lithuanian. Physicians who were on leave (e.g., maternity/paternity leave, medical leave) or working exclusively in institutions without contracts with the National Health Insurance Fund under the Ministry of Health did not participate in the survey. A total of 671 physicians participated in this survey (this sample represents 4.81% of Lithuanian physicians’ population).

Sample size calculations were performed assuming a 95% confidence level, a 5% margin of error, and a conservative prevalence estimate of 50%, with finite population correction applied separately for each professional group. The minimum required numbers of respondents were 265 5th- and 6th-year medical students (*n* = 846), 311 residents (*n* = 1614), and 374 physicians (*n* = 13,951). The final achieved sample comprised 497 students, 199 residents, and 671 physicians. This corresponds to margins of error of approximately ±2.8% for students, ±6.5% for residents, and ±3.7% for physicians. These values indicate that the estimates for physicians and students are precise, whereas the results for residents should be interpreted with more caution due to the smaller sample size.

A questionnaire was developed by the authors, drawing on the results of previously conducted studies and survey instruments used in other countries [8,24,25]. The development process was guided by theoretical frameworks on health workforce mobility, turnover intention, and push-pull migration dynamics. Items were adapted and formulated to reflect the Lithuanian health system context, capturing factors at the individual, institutional, and systemic levels. This approach ensured both comparability with international evidence and contextual relevance.

A pilot study was conducted with a convenience sample comprising 10 medical students, 5 residents, and 5 physicians (*n* = 20) to assess the clarity, comprehensibility, and face validity of the questionnaire. The feedback obtained from the pilot participants informed minor revisions, including refinement of item wording and response categories. These adjustments were undertaken to enhance the accuracy and contextual relevance of the instrument for the target study population. Data from the pilot test were not included in the final analysis.

The special questionnaire was applied in this survey and covered these topics:

1. Basic sociodemographic information of respondents—includes gender, age, marital status, number of children under 14 years of age, place of residence (urban/rural), study funding status (state-funded or self-funded), previous experience abroad (study or internship), and foreign language proficiency.
2. Intentions to work abroad—assess whether respondents are considering or planning to emigrate, the planned time frame (within 1–2 years, 2–3 years, after completing studies/residency, etc.), preferred destination countries, and the main reasons for this decision (e.g., higher salary, better living conditions, greater career opportunities, or dissatisfaction with the Lithuanian healthcare system).
3. Career plans within the healthcare system—explore respondents’ intentions to work in their chosen medical specialty after graduation or residency, their perceived job opportunities in Lithuania, and their attitudes toward potentially leaving the medical profession altogether, including reasons for such considerations (e.g., physical and psychological strain, low salary, or perceived unsuitability of the chosen field).

A two-step statistical analysis was conducted. First, descriptive statistics were calculated to summarize the characteristics of the study sample. Analyses were performed using SPSS Statistics (version 22.0; IBM Corp., Armonk, NY, USA) and Microsoft Excel 2019 (Microsoft Corp., Redmond, WA, USA). Continuous variables are reported as means with standard deviations mean (SD), and categorical variables as frequencies and percentages.

To examine associations between professional groups (medical students, residents, and physicians) and their intentions to migrate abroad, binary logistic regression analysis was applied using the Enter method, where all predictor variables were entered into the model simultaneously. Odds ratios (ORs) with corresponding 95% confidence intervals (95% CI) were calculated by exponentiating the regression coefficients, providing estimates of the relative likelihood of emigration intentions across groups.

A *p*-value < 0.05 was considered statistically significant for all analyses.

At the beginning of the questionnaire, participants were informed about the aim of the study, the voluntary nature of participation, data confidentiality, and their right to withdraw at any time. The survey was anonymous—no personally identifiable information was collected, and no participant tracking or follow-up was conducted. Therefore, signed informed consent was not required, the submission of the completed questionnaire was considered implied informed consent, in line with recognized ethical standards for anonymous online surveys. The study was conducted in accordance with the ethical principles outlined in the Declaration of Helsinki and was approved by the Lithuanian Bioethics Committee (Approval No. 6B-22-115).

## 3. Results

Most study participants were women, and most reported knowledge of more than one foreign language. The mean age of medical students was 23.60 years (SD = 1.46), residents—27.38 years (SD = 2.05), and physicians—44.53 years (SD = 13.40). Among physicians, 33.83% reported having children, as did 17.09% of residents; this figure was below 1.00% among students (0.81%). The main sociodemographic characteristics of participants are summarized in Table 1.

The largest group of participating physicians were family doctors (22.80%), followed by those licensed only for general medical practice (12.36%), internists (8.20%), and pediatricians (6.86%). In this study, physicians with only a general medical practice license are those who did not pursue further specialization. At least one response was received from nearly all medical specialties, except for dietetics, neurosurgery, and cardiac surgery.

Among residents, the most common specializations were family medicine (26.13%), emergency medicine (9.05%), and neurology (6.03%). All residents held a license to practice general medicine.

Since the medical students had not yet completed their studies, they were asked to indicate the specialization they planned to pursue. The most frequently mentioned fields were family medicine (11.27%), anesthesiology and intensive care (8.25%), psychiatry (6.84%), dermatovenerology (6.84%), obstetrics and gynecology (6.64%), and cardiology (6.64%). Other specializations were mentioned less frequently.

A total of 50.30% of medical students, 35.68% of residents, and 10.43% of physicians indicated that they were planning to leave Lithuania. Among residents, none had made a definitive decision to emigrate. Students most often associated their potential emigration with the completion of their studies, with 22.94% reporting plans to leave within the next 2–3 years. In all groups, the majority of those planning to emigrate stated that they intended to return to Lithuania, rather than leave for permanent employment abroad (Table 2).

To assess the influence of social factors on the intention to emigrate among students, residents, and physicians, a logistic regression analysis was performed. Among physicians, males were 1.81 times more likely to plan emigration compared to females, while increasing age was associated with a lower likelihood of emigration. Physicians who had completed non-state-funded medical studies were 3.2 times more likely to plan emigration. Additional foreign language learning was significantly associated with a higher likelihood of planning to work abroad across all three groups (*p* < 0.05). Among students, those who had previously gone abroad for internships or study purposes were more likely to express intentions to emigrate for work (Table 3).

The most specified reason for planning to leave Lithuania among medical students, residents, and physicians was higher salary. Better living conditions and greater professional opportunities were the second most frequently mentioned reasons. A notable proportion of medical students (44.40%) identified a lack of job opportunities matching their specialization, whereas this reason was reported by only 16.90% of residents and 2.86% of physicians, suggesting it was less relevant for these groups (Table 4).

Medical students, residents, and physicians were asked to indicate net monthly income (after taxes) that they would consider sufficient for a good quality of life. The mean amounts reported by students (€3154.72, SD = 1760.79) and residents (€3221.83, SD = 1038.58) were similar, while physicians indicated a higher amount (€4392.29, SD = 1925.63). The lowest amount was reported by students (€800.00), while the highest was €20,000.00, noted by both students and physicians. Among residents, the maximum reported amount was €8000.00. Across all groups, the most frequently mentioned amount was €3000.00.

The most reported barriers to potential emigration were language barriers (65.22%), separation from family or friends (55.24%), and difficulties related to adaptation and integration (48.08%). A large majority indicated a willingness to improve their foreign language skills (92.40% of students, 85.92% of residents, and 70.00% of physicians), attend additional professional training (66.24%), or accept positions below their current qualification level (19.95%).

The commonly indicated destination across all groups was Germany (57.20% of students, 33.80% of residents, and 37.14% of physicians). The second most frequent choice among students was Switzerland (46.00%), while both residents and physicians commonly mentioned Sweden as their second preference. The United Kingdom and Norway were also frequently selected (Table 5).

A significant proportion of physicians are currently considering (26.38%) or have previously considered (17.73%) leaving the medical profession. Among students, only 0.60% expressed doubt about working in their chosen field after graduation, while this proportion was 4.02% among residents. When combining those who plan to emigrate and those considering leaving the profession, there were 253 medical students (50.91%), 79 residents (39.70%), and 247 physicians (36.81%). This estimate for physicians includes only those who currently report considering leaving their medical specialty. The main reasons cited by students and residents for not planning to work in their specialty after graduation were, in order of priority: low salary, physical and psychological strain, and the realization that the field is not suitable for them. Among physicians, the most frequently mentioned reasons were physical and psychological strain, low salary, and administrative burden.

To more accurately assess the risk of attrition or emigration among currently practicing professionals (residents and physicians), only the strongest indicators were included in the analysis—those who selected statements such as “concrete, planned or ongoing actions”, indicated a specific departure timeframe, or answered “yes, I am currently considering leaving the profession”.

Among residents and physicians, the highest number of those planning to leave the workforce or emigrate were in the field of family medicine—4 residents and 62 physicians. A notable number of physicians were also from the fields of general medical practice (41), pediatrics (19), physical medicine and rehabilitation (17), and internal medicine (13). Some physicians held more than one specialty; therefore, a single physician’s departure could be counted under multiple specialties. The total number of specialties reported among physicians was 826 (Appendix A Table A1, Table 6).

Respondents were asked to indicate which professional status they associate themselves with, according to definitions currently valid in Lithuania. The majority identified as practicing medical professionals—defined as persons working in direct clinical care with patients [27]: 304 students (60.92%), 142 residents (71.36%), and 473 physicians (70.49%). A smaller proportion identified as professionally active medical professionals–defined as persons working in healthcare, science education, or management, whose role requires or benefits from medical education [27]: 189 students (37.86%), 56 residents (28.14%), and 183 physicians (27.27%). The remaining respondents did not indicate status. These results suggest that a substantial share of current and future healthcare professionals working within the system may not be directly involved in clinical care. This could contribute to extended care time for services and an apparent shortage of clinical staff.

Slightly more than a half of all residents (50.75%) reported holding an additional job besides their residency duties, most of them (95.05%) working as general medical practitioners. Among physicians, 44.11% reported working in more than one healthcare institution. Additionally, 38.30% of physicians and 19.10% of residents indicated that at least one of their workplaces was a private healthcare institution.

## 4. Discussion

According to an analysis conducted by the Government Strategic Analysis Centre in 2021, Lithuania is expected to face a shortage of 2799 healthcare professionals with various qualifications by the year 2030, assuming that part of the workforce will be requalified or take over tasks from other specialists facing shortages. However, under a pessimistic scenario—when professionals are unwilling or unable to take on additional roles and instead leave the healthcare sector—the shortage could increase to 5400 specialists [28].

The findings of this study reveal a significant intention among medical students, residents, and physicians in Lithuania to emigrate or leave the profession. This trend is not unique to Lithuania but is consistent with broader European and global patterns of healthcare worker migration, which pose serious challenges to the stability and accessibility of healthcare systems [5,8].

Our study found that as many as 50.30% of medical students, 35.68% of residents, and 10.43% of physicians plan to leave Lithuania. A similar study conducted in Croatia reported that 34.60% of medical students intended to emigrate [29], while in Romania the figure was 42.17% [30]. When compared to previous studies conducted in Lithuania, the results also show variation. In a 2002 study conducted by M. Stankūnas et al. prior to Lithuania’s accession to the European Union, 60.70% of residents and 26.00% of physicians planned to emigrate [31]. In a 2018 study by B. Goštautienė et al., 39.00% of students, 21.00% of residents, and 6.00% of physicians reported plans to leave [13]. These differences may be partly explained by external factors: the first study was conducted before Lithuania joined the EU, while the second preceded the COVID-19 pandemic.

Germany was the most frequently mentioned emigration destination among students, residents, and physicians in our study (Table 4), which is consistent with findings from other international studies [29,30].

Although the results of our study are cause for concern, data from the Education Management Information System show that within 12 months after graduation, the average number of medical students who officially declared emigration was 33.56 (7.24%) and 8.78 (2.30%) among residents, relative to all program graduates [32]. These figures align with the share of respondents in our study who reported a firm decision to emigrate: 38 students (7.64%) and 13 residents and physicians (1.94%) [Appendix A, Table A2].

The migration of healthcare professionals is often driven by a combination of factors; however, the main push factors continue to be insufficient salaries, poor working conditions, limited opportunities for professional development, and excessive workloads. In contrast, pull factors include higher incomes, better living conditions, and improved career prospects in other countries [10,33]. Similar drivers were identified in our study (Table 4), and their relevance is consistent with the broader context of the European region [34,35].

Between 2015 and 2023, mean monthly salaries (before tax) for medical graduates and residents (both measured 12 months after graduation) increased by a factor of 5.96, from €490.31 to €2919.89, and by a factor of 4.12, from €1578.48 to €6502.79, respectively [Appendix A Table A2]. Nevertheless, this sharp increase remains insufficient when compared with the mean salary expectations expressed by students, residents, and physicians. A significant salary gap between countries may remain a key factor contributing to physician migration [36,37].

It is particularly important to note that the majority of respondents intending to emigrate plan to work in their chosen medical specialty abroad, rather than changing to another field. This means that their emigration directly contributes to the shortage of healthcare professionals in Lithuania [38]. This finding is consistent with other studies, which have shown that professional migration often leads to long-term challenges in the quality and accessibility of healthcare services [11,12].

Early-career respondents—particularly medical students and residents—were markedly more likely to express intentions to emigrate than physicians, while only a very small proportion considered leaving the medical profession altogether. In contrast, physicians were much more likely to report currently or previously considering leaving their specialty. This pattern suggests that the emigration intentions of students and residents may reflect uncertainty and career exploration typical of the transition from education to practice, whereas physicians’ intentions to leave the profession are more often associated with long-term workload and job-related disillusionment. Such differences are consistent with previous studies showing that migration intentions are more common at early career stages, whereas intentions to leave the profession tend to emerge later in the professional pathway [19,22].

The main potential drivers of emigration across all groups were financial: higher expected salaries, better living conditions, and broader professional opportunities abroad. However, students additionally emphasized uncertainty about finding employment in their chosen specialty, which was far less relevant to residents or physicians. By contrast, physicians considering leaving the profession most often cited physical and psychological strain, low salary, and administrative burden, while students and residents mentioned low salary, workload, and doubts about their chosen field. These patterns are consistent with earlier evidence indicating that financial and working-condition factors are the dominant push factors among early-career professionals, whereas later-career physicians are more affected by burnout and systemic pressures [21,23].

These findings highlight the need to apply retention strategies tailored to different career stages. At the national level, policy measures should focus on ensuring competitive remuneration, providing access to positions aligned with graduates’ specialties, and reducing excessive workloads for early-career professionals. At the European Union level, coordinated measures are needed to balance workforce mobility and prevent the disproportionate outflow of healthcare professionals from Central and Eastern European countries, including Lithuania [18,39]. At the institutional level, universities and healthcare institutions could strengthen mentorship systems, provide psychological support to reduce burnout, and create clearer career development pathways, particularly in specialties identified as having the highest risk of departure—such as family medicine, general practice, pediatrics, physical medicine and rehabilitation, and internal medicine.

In the Lithuanian context, our study updates earlier data indicating similar migration trends, while also including a broader sample of participants and an analysis of the main reasons behind these intentions [13]. These insights provide valuable information for policymakers aiming to develop effective strategies to retain healthcare professionals. Such strategies may include increasing salaries, reducing workloads, improving opportunities for professional development, and strengthening psychological support systems [40,41].

Nevertheless, our study has certain limitations that should be acknowledged. First, it employed a cross-sectional design, which allows for the assessment of participants’ intentions at a single point in time. As a result, it is not possible to observe changes over time or determine whether stated intentions to migrate or leave the medical profession translated into actual actions. Longitudinal studies would be needed to confirm such behavioral outcomes.

The second limitation relates to the research instrument. The questionnaire used in this study was developed specifically for the purposes of this research, as no standardized and validated tools were identified that could adequately measure migration and career exit intentions among medical students, residents, and physicians in Lithuania. Although the questionnaire was developed based on a comprehensive review of the literature and expert consultations and was pilot-tested to assess its clarity, structure, and relevance, it has not been validated internationally. This may limit the comparability of findings with similar studies conducted in other countries.

The third limitation concerns the varied survey distribution strategies applied to different respondent groups. Medical students were reached both via institutional email and in-person during lectures, residents were contacted via institutional email and through representatives, while physicians received the survey through healthcare institutions. This mixed approach, relying on intermediaries and voluntary participation, may introduce an unknown degree of selection bias and limit the generalizability of the results to the broader target population.

It is also important to note that the study was conducted during the COVID-19 pandemic—an unprecedented period of stress and disruption in the healthcare sector. Therefore, the responses from residents and physicians, in particular, should be interpreted in the context of the pandemic’s potential influence on career planning and professional intentions.

Despite these limitations, the study also has notable strengths. The sample size was large (*n* = 1367) and nationally representative, including 5th and 6th year medical students, residents, and physicians. In both the student and physician groups, the number of respondents exceeded the minimum required for robust analysis. Furthermore, the inclusion of respondents at three distinct stages of the medical career pathway (student, resident, and physician) provides a unique opportunity to assess intentions and attitudes across the professional lifecycle. Unlike many previous studies, this research examines not only migration intentions but also intentions to leave the medical profession altogether, offering a more comprehensive view of health workforce attrition.

Moreover, the COVID-19 pandemic highlighted the critical role of healthcare workers and exposed persistent issues related to their working conditions. This prompted a portion of professionals to reconsider their career paths and contributed to intentions to leave the medical profession [7,42]. These developments further underscore the need for continuous monitoring and assessment of migration and attrition trends among physicians, along with the factors influencing them.

In summary, our study confirms that migration and withdrawal from the medical profession are complex processes influenced not only by economic factors, but also by organizational and psychosocial aspects. Future research should focus on exploring individual motivations in greater depth, assessing the long-term impact of migration on the healthcare system, and evaluating the effectiveness of potential interventions [6].

## 5. Conclusions

Our study highlights that, when forecasting the supply and demand of healthcare human resources and shaping workforce policies, it is essential to consider not only objective data but also the subjective perspectives of current and future physicians. The findings revealed clear differences across career stages: medical students and residents were significantly more likely to express intentions to emigrate, whereas physicians more often reported considering leaving the profession. This suggests that migration intentions among early-career professionals may be driven by uncertainty and career exploration, while career exit intentions among physicians are more often related to long-term workload, burnout, and systemic challenges.

Addressing the main push factors identified in this study—low salaries, high workloads, limited professional opportunities, and psychological strain—requires retention strategies tailored to different career stages. At the national level, policies should focus on ensuring competitive remuneration, securing opportunities for employment aligned with graduates’ specialties, and reducing excessive workloads. At the European Union level, coordinated measures are needed to balance workforce mobility and prevent the disproportionate outflow of healthcare professionals from Central and Eastern European countries, including Lithuania. At the institutional level, universities and healthcare institutions should strengthen mentorship systems, provide psychological support, and establish clear career development pathways, particularly in specialties with the highest risk of attrition.

The problems identified in this study, supported by empirical data, may be relevant not only for Lithuanian policymakers but also for healthcare systems in other countries facing similar challenges. Therefore, it is essential to conduct such studies regularly, especially after major policy decisions that directly affect healthcare professionals, and to complement them with longitudinal research assessing whether stated intentions translate into actual behavior.

These findings should be interpreted in the context of Lithuania’s broader health system transformation following its independence in the early 1990s. Unlike many other former Soviet Union countries, Lithuania—together with the other Baltic States—pursued a distinct path shaped by its accession to the European Union in 2004, transitioning from a centrally planned Semashko model to decentralized management and diversified funding. While these reforms expanded patient choice and created new organizational opportunities, persistent challenges such as inadequate remuneration, heavy workloads, and migration pressures continue to affect workforce retention, professional well-being, and physicians’ long-term career intentions.

## Figures and Tables

**Table 1:** Sociodemographic characteristics of the medical students, residents, and physicians who participated in the study.

Sociodemographic Characteristics	Medical Students	Medical Residents	Physicians
**Gender, *n* (%)**	***n* = 497**	***n* = 199**	***n* = 668**
Male	375 (75.45)	155 (77.89)	518 (77.55)
Female	122 (24.55)	44 (22.11)	150 (22.46)
**Age group, *n* (%)**	***n* = 489**	***n* = 199**	***n* = 669**
20–29	485 (99.18)	170 (85.43)	82 (12.26)
30–39	4 (0.82)	29 (14.57)	234 (34.98)
40–49	-	-	96 (14.35)
50–59	-	-	137 (20.48)
60–69	-	-	104 (15.55)
70+	-	-	16 (2.39)
**Marital status, *n* (%)**	***n* = 497**	***n* = 198**	***n* = 671**
Single	198 (39.84)	44 (22.22)	130 (19.37)
Married	9 (1.81)	72 (36.36)	448 (66.77)
In a relationship (not married)	290 (58.35)	82 (41.42)	93 (13.86)
**Children under 14 years, *n* (%)**	***n* = 494**	***n* = 198**	***n* = 667**
One	3 (0.61)	22 (11.11)	115 (17.24)
Two or more	1 (0.20)	12 (6.06)	112 (16.79)
None	490 (99.19)	164 (82.83)	440 (65.97)
**Foreign language proficiency (excluding Lithuanian), *n* (%)**	***n* = 495**	***n* = 198**	***n* = 668**
One	228 (46.06)	84 (42.42)	178 (26.65)
Two	202 (40.81)	78 (39.39)	363 (54.34)
Three or more	65 (13.13)	35 (18.18)	95 (19.01)
**Language(s) spoken, *n* (%)**	***n* = 495**	***n* = 198**	***n* = 668**
English	493 (99.60)	197 (99.50)	577 (86.38)
Russian	159 (32.12)	86 (43.43)	515 (77.10)
German	114 (23.03)	36 (18.18)	96 (14.37)
French	30 (6.06)	8 (4.04)	31 (4.64)
Other	30 (6.06)	21 (10.61)	104 (15.57)
Scandinavian (Norwegian, Danish, Swedish)	13 (2.63)	1 (0.51)	17 (2.55)

**Table 2:** Opinions of students, residents, and physicians regarding emigration for work abroad.

	Medical Students (*n* = 497)	Medical Residents (*n* = 199)	Physicians (*n* = 671)
**Planning to emigrate, *n* (%)**			
Yes	250 (50.30)	71 (35.68)	70 (10.43)
No	247 (49.70)	128 (64.32)	601 (89.57)
**Certainty of decision, *n* (%)**			
Final decision	38 (7.64)	0 (0.00)	13 (1.94)
Specific, planned or ongoing actions	56 (11.27)	10 (5.03)	25 (3.73)
Consideration without specific actions	117 (23.54)	38 (19.10)	20 (2.98)
Preliminary intentions/plans	39 (7.85)	23 (11.56)	12 (1.79)
**Planned timeframe for emigration, *n* (%)**			
Within the next 12 months	36 (7.24)	1 (0.50)	13 (1.93)
Within the next 24–36 months	114 (22.94)	24 (12.06)	24 (3.58)
Within the next 48–60 months	46 (9.26)	18 (9.05)	21 (3.13)
After more than 60 months	38 (7.65)	15 (7.54)	12 (1.79)
Not specified	16 (3.22)	13 (6.53)	0 (0.00)
**Planned duration of emigration, *n* (%)**			
Up to 12–24 months	14 (2.82)	6 (3.02)	4 (0.60)
Up to 36–48 months	38 (7.65)	16 (8.04)	11 (1.64)
More than 60 months	105 (21.13)	20 (10.05)	30 (4.47)
Permanent (no intention to return)	72 (14.49)	15 (7.54)	25 (3.73)
Not specified	21 (4.23)	14 (7.04)	0 (0.00)

**Table 3:** Social factors related to the risk of emigration for work abroad among medical students, residents, and physicians.

Characteristic	Medical Students OR (95% CI)	Medical Residents OR (95% CI)	Physicians OR (95% CI)
Gender (male)	1.18 (0.78–1.79)	1.33 (0.65–2.73)	1.81 (1.06–3.10) *
Age (years)	1.06 (0.92–1.22)	0.96 (0.84–1.11)	0.95 (0.92–0.97) *
Foreign language learning	4.89 (2.59–9.25) *	2.58 (1.19–5.56) *	11.19 (3.47–36.07) *
State-funded medical studies	0.92 (0.64–1.32)	0.98 (0.52–1.83)	0.31 (0.16–0.58) *
Having family or friends abroad	1.40 (0.92–2.11)	1.09 (0.58–2.05)	1.43 (0.84–2.44)
Having children under 14 years of age	2.98 (0.31–28.81)	0.51 (0.22–1.19)	1.30 (0.78–2.17)
Experience abroad for clinical placement or study (during studies)	2.96 (1.94–4.51) *	1.81 (0.98–3.33)	-

* *p* < 0.05; OR—odds ratio; 95% CI—95% confidence interval.

**Table 4:** Reasons for planning to emigrate among medical students, residents, and physicians.

Reason, *n* (%)	Medical Students (*n* = 250)	Medical Residents (*n* = 71)	Physicians (*n* = 70)
Higher salary	208 (83.20)	47 (66.20)	35 (50.00)
Better living conditions	189 (75.60)	40 (56.34)	15 (21.43)
More professional opportunities	181 (72.40)	40 (56.34)	15 (21.43)
Lack of job opportunities in chosen specialization	111 (44.40)	12 (16.90)	2 (2.86)
Offer from an institution abroad	21 (8.40)	8 (11.27)	3 (4.29)
Family-related reasons (to join relatives)	16 (6.40)	7 (9.86)	1 (1.43)
Better work culture	7 (2.80)	3 (4.23)	3 (4.29)
Healthcare system (e.g., political decisions, excessive bureaucracy)	4 (1.60)	2 (2.82)	4 (5.71)
Residency issues (e.g., tuition fees, not accepted to preferred program)	7 (2.80)	0 (0.00)	1 (1.43)
Other	13 (5.20)	5 (7.04)	2 (2.86)
Not specified	18 (7.20)	11 (15.49)	1 (1.43)

**Table 5:** Most frequently indicated emigration destinations among medical students, residents, and physicians.

Country, *n* (%)	Medical Students (*n* = 250)	Medical Residents (*n* = 71)	Physicians (*n* = 70)
Germany	143 (57.20)	24 (33.80)	26 (37.14)
Switzerland	115 (46.00)	15 (21.13)	10 (14.29)
Scandinavian countries (Denmark, Norway, Sweden)	98 (39.20)	33 (46.48)	51 (72.86)
United Kingdom	47 (18.80)	11 (15.49)	17 (24.29)
Austria	46 (18.40)	2 (2.82)	4 (5.71)
North America (USA, Canada)	34 (13.60)	9 (12.68)	6 (8.57)
France	21 (8.40)	5 (7.04)	6 (8.57)
Other	77 (30.80)	26 (36.62)	33 (47.14)
Not specified	18 (7.20)	5 (7.04)	3 (4.29)

**Table 6:** Specialties reported by residents and physicians with concrete intentions to emigrate or leave the profession.

Specialties *	Medical Residents (*n* = 199)	Physicians (*n* = 826)
Medical doctor’s practice	199	41
General practitioners (GPs)	4	62
Internal medicine	2	39
Pediatrics	0	46
Dietetics	0	n.a.
Surgery	1	12
Orthopedics traumatology	0	2
Pediatric surgery	0	0
Anesthesiology reanimation	0	6
Obstetrics gynecology	0	5
Otorhinolaryngology	0	4
Psychiatry	1	13
Genetics	0	0
Forensic medicine	0	0
Physical medicine and rehabilitation	1	20
Pathology	0	3
Laboratory medicine	n.a.	1
Radiology	0	5
General dental practice	n.a.	0
Other (not included in specialties and subspecialties list)	1	4
Total, *n* (%)	10 (5.03)	264 (31.96)

* The list of specialties is based on the classification approved by the Ministry of Health of the Republic of Lithuania [26]; n.a.—data not available due to non-response. Note: The figures represent the number of reported specialties, not individual respondents. Some physicians held multiple specialties and therefore may be counted more than once. All residents held a medical doctor‘s license.

## Data Availability

Data is contained within the article.

## Appendix A

**Table A1:** Specialties and subspecialties reported by residents and physicians with concrete intentions to emigrate or leave the profession.

Specialties Subspecialties *	Medical Residents (*n* = 199)	Physicians (*n* = 826)
**Medical doctor’s practice**	**199**	**41**
**General practitioners (GPs)**	**4**	**62**
**Internal medicine**	**2**	**39**
internal medicine	1	13
allergology and clinical immunology	0	0
occupational medicine	n.a.	0
dermatovenerology	0	1
endocrinology	n.a.	2
gastroenterologist	0	2
geriatrics	0	4
hematology	0	0
infectiology	0	2
cardiology	0	2
nephrology	n.a.	6
neurology	1	2
pulmonology	0	2
rheumatology	n.a.	0
clinical toxicology	n.a.	1
oncology chemotherapy	0	0
oncology radiotherapy	n.a.	2
intensive care medicine	n.a.	n.a.
**Pediatrics**	**0**	**46**
pediatrics	0	19
neonatology	n.a.	1
pediatrics and pediatric endocrinology	n.a.	1
pediatrics and pediatric gastroenterology	n.a.	2
pediatrics and pediatric hematology	0	1
pediatrics and pediatric cardiology	n.a.	4
pediatrics and pediatric nephrology	0	3
pediatrics and pediatric neurology	0	2
pediatrics and neonatology	0	1
pediatrics and pediatric pulmonology	n.a.	2
pediatrics and pediatric infectious diseases	n.a.	3
pediatrics and pediatric rheumatology	0	2
pediatrics and pediatric allergology	n.a.	0
pediatrics and pediatric intensive care medicine	0	5
**Dietetics**	**0**	**n.a.**
**Surgery**	**1**	**12**
surgery	0	6
abdominal surgery	1	1
plastic and reconstructive surgery	0	0
vascular surgery	0	0
heart surgery	0	n.a.
neurosurgery	0	n.a.
chest surgery	n.a.	1
urology	0	2
**Orthopedics traumatology**	**0**	**2**
**Pediatric surgery**	**0**	**0**
**Anesthesiology reanimation**	**0**	**6**
**Obstetrics gynecology**	**0**	**5**
**Otorhinolaryngology**	**0**	**4**
**Psychiatry**	**1**	**13**
psychiatry	1	8
child and adolescent psychiatry	0	5
**Genetics**	**0**	**0**
**Forensic medicine**	**0**	**0**
**Physical medicine and rehabilitation**	**1**	**20**
physical medicine and rehabilitation	1	17
sports medicine	n.a.	3
**Pathology**	**0**	**3**
**Laboratory medicine**	**n.a.**	**1**
**Radiology**	**0**	**5**
**General dental practice**	**n.a.**	**0**
**Other (not included in specialties and subspecialties list)**	**1**	**4**
clinical pharmacology	n.a.	2
emergency medical doctor	1	2
**Total, *n* (%)**	**10 (5.03)**	**264 (31.96)**

* The list of specialties and subspecialities is based on the classification approved by the Ministry of Health of the Republic of Lithuania [26]; n.a.—data not available due to non-response. Note: The figures represent the number of reported specialties, not individual respondents. Some physicians held multiple specialties and therefore may be counted more than once. All residents held a medical doctor‘s license.

**Table A2:** Employment data of medical students and residents 12 months after graduation [32].

Years	2015	2016	2017	2018	2019	2020	2021	2022	2023	2015–2023
**Number of graduates, *n* ***	**821**	**845**	**850**	**863**	**825**	**855**	**849**	**848**	**850**	**845.11**
students, *n* (%)	446 (54.32)	427 (50.53)	486 (57.18)	487 (56.43)	459 (55.64)	481 (56.26)	463 (54.53)	464 (54.72)	434 (51.06)	460.78 (54.52)
residents, *n* (%)	375 (45.68)	418 (49.47)	364 (42.82)	376 (43.57)	366 (44.36)	374 (43.74)	386 (45.47)	384 (45.28)	416 (48.94)	384.33 (45.48)
**Declared emigration, *n* (%) ***	**31 (3.78)**	**43 (5.09)**	**63 (7.41)**	**61 (7.07)**	**41 (4.97)**	**34 (3.98)**	**36 (4.24)**	**39 (4.60)**	**33 (3.88)**	**42.33 (5.00)**
Students **	25 (5.61)	26 (6.09)	48 (9.88)	48 (9.86)	27 (5.88)	27 (5.61)	35 (7.56)	35 (7.54)	31 (7.14)	33,56 (7.24)
Residents ***	6 (1.60)	17 (4.07)	15 (4.12)	13 (3.46)	14 (3.83)	7 (1.87)	1 (0.26)	4 (1.04)	2 (0.48)	8.78 (2.30)
**Employment service, *n* (%) ***	**7 (0.85)**	**4 (0.47)**	**15 (1.76)**	**23 (2.67)**	**19 (2.30)**	**30 (3.51)**	**15 (1.77)**	**14 (1.65)**	**16 (1.88)**	**15.89 (1.87)**
Students **	6 (1.35)	3 (0.70)	14 (2.88)	21 (4.31)	15 (3.27)	26 (5.41)	9 (1.94)	12 (2.59)	13 (3.00)	13.22 (2.83)
Residents ***	1 (0.27)	1 (0.24)	1 (0.27)	2 (0.53)	4 (1.09)	4 (1.07)	6 (1.55)	2 (0.52)	3 (0.72)	2.67 (0.70)
**Employed, *n* (%) ***	**745 (90.74)**	**754 (89.23)**	**734 (86.35)**	**744 (86.21)**	**724 (87.76)**	**761 (89.01)**	**771 (90.81)**	**768 (90.57)**	**780 (91.76)**	**753.44 (89.16)**
Students **	383 (85.87)	364 (85.25)	391 (80.45)	393 (80.70)	381 (83.01)	401 (83.37)	393 (84.88)	394 (84.91)	371 (85.48)	385.67 (83.77)
Residents ***	362 (96.53)	390 (93.30)	343 (94.23)	351 (93.35)	343 (93.72)	360 (96.25)	378 (97.93)	374 (97.40)	409 (98.32)	367,78 (95.67)
**Among those employed: working in their field of qualification, *n* (%)**	**709 (95.17)**	**720 (94.49)**	**705 (96.05)**	**691 (92.88)**	**691 (95.44)**	**719 (94.48)**	**726 (94.16)**	**719 (93.62)**	**732 (93.85)**	**712.44 (94.57)**
Students **	370 (82.96)	355 (83.14)	376 (77.37)	367 (75.36)	370 (80.61)	378 (78.59)	373 (80.56)	373 (80.39)	354 (81.57)	368.44 (80.06)
Residents ***	339 (90.40)	365 (87.32)	329 (90.38)	324 (86.17)	321 (87.70)	341 (91.18)	353 (91.45)	346 (90.10)	378 (90.87)	344.00 (89.51)
**Average gross monthly income under employment contract (before tax), EUR.**	-	-	-	-	-	-	-	-	-	-
students	490.31	546.17	693.12	1669.88	2225.86	2481.98	2241.54	2558.3	2919.89	1758.56
residents	1578.48	1740.07	2184.55	3182.94	4042.96	4287.09	4999.42	5897.41	6502.79	3823.97

*—from all graduates (including students with the citizenship of the Republic of Lithuania and all residents). **—from all medical students who held the citizenship of the Republic of Lithuania. ***—from all medical residents.
